# Optics for coherent X-ray applications

**DOI:** 10.1107/S1600577514016415

**Published:** 2014-08-27

**Authors:** Makina Yabashi, Kensuke Tono, Hidekazu Mimura, Satoshi Matsuyama, Kazuto Yamauchi, Takashi Tanaka, Hitoshi Tanaka, Kenji Tamasaku, Haruhiko Ohashi, Shunji Goto, Tetsuya Ishikawa

**Affiliations:** a RIKEN SPring-8 Center, Kouto 1-1-1, Sayo, Hyogo 679-5148, Japan; b Japan Synchrotron Radiation Research Institute (JASRI), Kouto 1-1-1, Sayo, Hyogo 679-5198, Japan; c Department of Precision Engineering, Graduate School of Engineering, The University of Tokyo, Hongo 7-3-1, Bunkyo-ku, Tokyo 113-8656, Japan; d Department of Precision Science and Technology, Graduate School of Engineering, Osaka University, 2-1 Yamada-oka, Suita, Osaka 565-0871, Japan

**Keywords:** X-ray optics, X-ray beamline, coherence

## Abstract

Developments of X-ray optics for full utilization of diffraction-limited storage rings (DLSRs) are presented. The expected performance of DLSRs is introduced using the design parameters of SPring-8 II. To develop optical elements applicable to manipulation of coherent X-rays, advanced technologies on precise processing and metrology were invented. With propagation-based coherent X-rays at the 1 km beamline of SPring-8, a beryllium window fabricated with the physical-vapour-deposition method was found to have ideal speckle-free properties. The elastic emission machining method was utilized for developing reflective mirrors without distortion of the wavefronts. The method was further applied to production of diffraction-limited focusing mirrors generating the smallest spot size in the sub-10 nm regime. To enable production of ultra-intense nanobeams at DLSRs, a low-vibration cooling system for a high-heat-load monochromator and advanced diagnostic systems to characterize X-ray beam properties precisely were developed. Finally, new experimental schemes for combinative nano-analysis and spectroscopy realised with novel X-ray optics are discussed.

## Abstract 2

Developments of optics for coherent X-ray applications and their role in diffraction-limited storage rings are described.

## 1. Introduction   

A diffraction-limited storage ring (DLSR), which combines an ultra-low-emittance storage ring with undulators, is capable of generating X-ray beams with extremely high average brilliance and a high degree of transverse coherence. A round collimated X-ray beam from a DLSR facilitates the generation of an intense small X-ray spot in the nanometre regime by using focusing optics, which expands the applicable targets of major X-ray techniques, such as diffraction, scattering, spectroscopy and imaging, to highly complex materials that cannot be analysed with existing probes. Furthermore, high transverse coherence enhances capabilities of coherence-related applications, such as single-particle coherent imaging (Miao *et al.*, 1999 ▶), ptychography (Rodenburg *et al.*, 2007 ▶) and correlation spectroscopy (Sutton, 2008 ▶). High transverse coherence in combination with advanced high-speed two-dimensional detectors will allow time-tracing measurements for spontaneously fluctuating systems with a timescale down to the nanosecond regime. These properties of DLSRs are complementary to those of X-ray free-electron lasers (XFELs), which are more suitable for single-shot destructive measurements with ultra-intense femtosecond X-ray pulses.

Full utilization of DLSRs requires development of beamline instrumentation including X-ray optics, sample manipulators with environmental controllers, detectors and data acquisition (DAQ) systems. In particular, X-ray optical systems used for monochromatization, focusing, collimation and polarization control are increasingly important for advanced applications. When using coherent X-rays from DLSRs, it is critical to achieve high quality for every optical element in order to preserve a coherent wavefront and to suppress unwanted speckles. Furthermore, X-ray optical systems are useful for diagnostics of radiation characteristics (*e.g.* source size, transverse coherence, wavefront, energy spectrum and pulse duration) and stability, which are required for performing advanced experiments with useful feedback for accelerator operations. These developments are important steps in the development of XFEL laser oscillators (Kim *et al.*, 2008 ▶), which requires ultra-precise control of both X-rays and the electron beam in six-dimensional phase space.

In this article we review developments of X-ray optics aiming for full utilization of DLSRs. In §2, we introduce the expected performance of a DLSR by using the tentative design parameters of SPring-8 II to facilitate the understanding of DLSRs. In §3, we summarize the developments of optical elements for coherent applications. §4 covers stability issues and advanced diagnostics. Finally, §5 introduces new experimental schemes achieved with innovative X-ray optics and DLSRs.

## 2. Performance of DLSRs   

In this section we introduce the expected performance of DLSRs by using the tentative design parameters of SPring-8 II, which is a project to upgrade the existing SPring-8 facility. SPring-8 II aims to generate brilliant X-rays from a storage ring with an ultra-low emittance of ∼100 pm rad, while maintaining high stability and reliability at reduced power consumption. To decrease emittance, we designed a new unit cell with a penta-bend achromat structure, which includes four bending magnets with segmented dipole field strengths. The beam energy will be reduced from 8 to 6 GeV. The design parameters are shown in Table 1 ▶. In order to minimize the shutdown period and reconstruction costs, we imposed constraints to utilize the existing tunnel and to retain the existing beam axes of the undulator beamlines.

**Figure 1:**
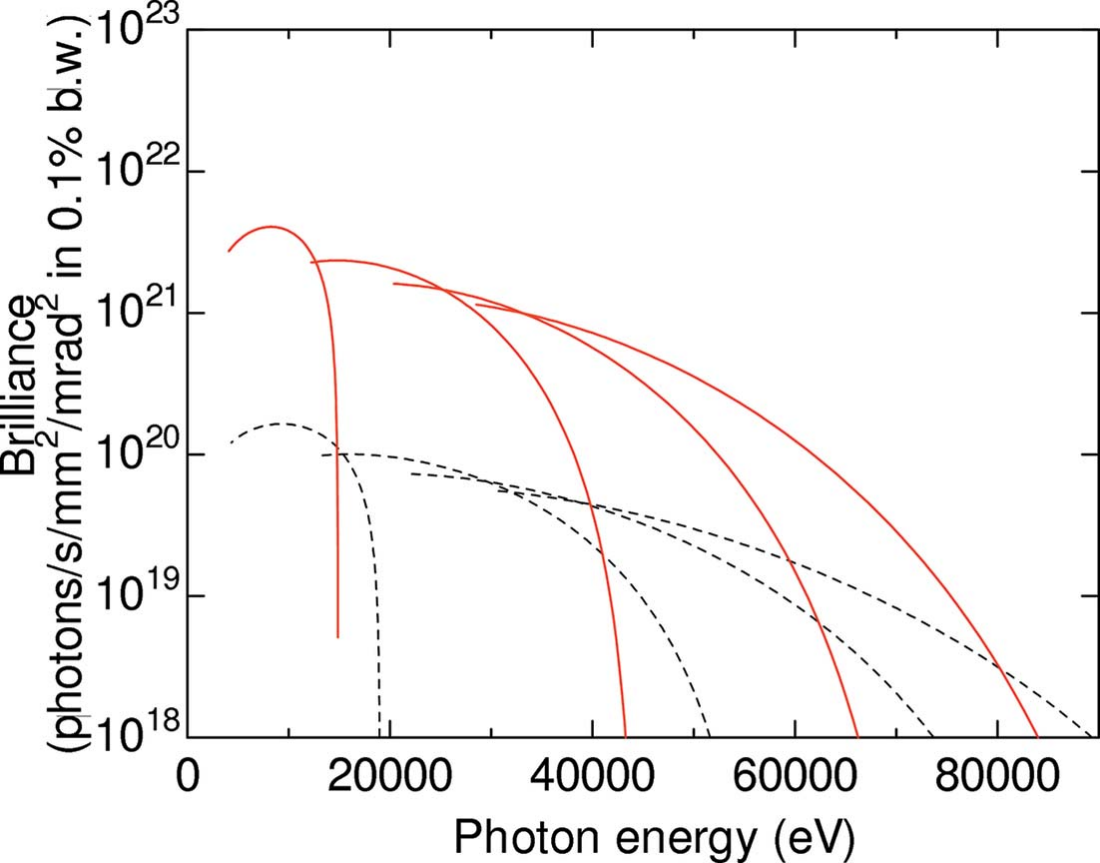
Comparison of brilliances for SPring-8 (black dashed curves) and SPring-8 II (red solid curves). Undulator parameters for SPring-8: period λ_u_ = 32 mm, number of periods *N* = 141 and total length *L*_u_ = 4.5 m. Undulator parameters for SPring-8 II: λ_u_ = 23 mm, *N* = 156 and *L*_u_ = 3.6 m. Maximum *K* value is 2.3.

**Figure 2:**
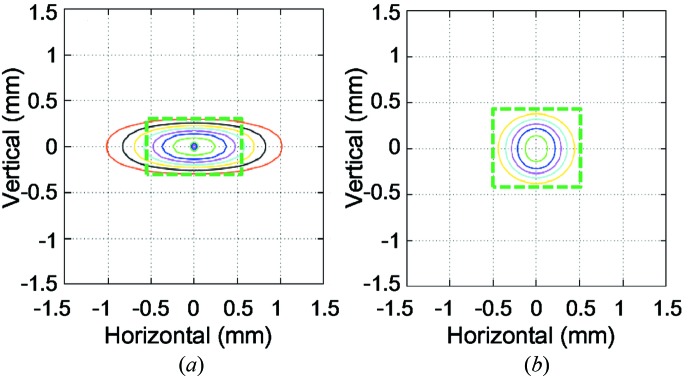
Typical beam profiles at 30 m distance from the source, simulated for (*a*) SPring-8 and (*b*) SPring-8 II. The rectangles show typical aperture sizes of the pre-slits to the first optics. The maximum heat loads through the apertures at a stored beam current of 100 mA are: (*a*) 340 W for a slit size of 1.1 (H) mm × 0.6 (V) mm, and (*b*) 200 W for 1.0 (H) mm × 0.8 (V) mm.

To produce high photon energies with 6 GeV operation, we plan to use short-period in-vacuum undulators. Fig. 1 ▶ shows brilliance as a function of photon energy for an undulator with a period length λ_u_ of 23 mm. In this case, one can keep the maximum *K* parameter over 2.3, which continuously covers a wide wavelength range by combining odd harmonics, with a minimum gap of 5 mm. We expect an increase of brilliance by a factor of 20, as shown in Fig. 1 ▶. Note that the total undulator length *L*_u_ decreases from 4.5 m to 3.6 m due to constraints in the new lattice design. Figs. 2(*a*) and 2(*b*) ▶ show a comparison of the beam profiles at the first optics, located 30 m from the source. The horizontal beam size for the new design is more concentrated due to the small horizontal emittance. This characteristic helps to reduce the heat load on the first optical element, because one can use a smaller aperture size for a pre-slit to the first optics without decreasing the photon flux. In the present case, an aperture size of 1.0 (H) mm × 0.8 (V) mm for the pre-slit is large enough to accommodate the central cone of the X-ray beam, which suppresses the maximum heat load below 200 W at a stored current of 100 mA. The photon flux with a Si(111) double-crystal monochromator (DCM) reaches ∼10^14^ photons s^−1^ for a photon energy range below 15 keV, which is ∼50% higher than that typically observed in the existing SPring-8 beamlines.

As shown in Table 1 ▶, the source size of SPring-8 II is as small as 24.0 (H) µm × 5.6 (V) µm (r.m.s.). This facilitates the generation of intense nano-beams by using a focusing optics scheme that directly demagnifies the source without a small aperture used as a secondary virtual source. In fact, production of a small spot of 230 (H) nm × 120 (V) nm is possible while keeping the original flux of ∼10^14^ photons s^−1^, which corresponds to a flux density as high as ∼10^21^ photons s^−1^ mm^−2^. In this estimation, we assumed the use of reflective focusing mirrors in the Kirkpatrick–Baez (KB) geometry (Kirkpatrick & Baez, 1948 ▶), located 74 m from the source with focal lengths of 0.3 m and 0.7 m. To achieve this fluence requires sufficient suppression of the vibration of each optical component, as discussed in §4.

## 3. X-ray optics with wavefront preservation   

High-quality optical elements are required for preserving the coherent wavefront from DLSRs and for suppressing unwanted speckles. In this section we review the developments of optical elements for coherent applications, mainly conducted at the coherent X-ray optics beamline BL29XUL of SPring-8 with a beamline length as long as 1 km (Tamasaku *et al.*, 2001 ▶; Ishikawa *et al.*, 2001 ▶). We first introduce the beamline, and then describe developments of Be windows, reflective mirrors and diamond crystals.

### 3.1. The 1 km beamline at SPring-8   

Since the horizontal emittance is much larger than the diffraction-limited condition at existing synchrotron sources, the degree of transverse coherence is insufficient to characterize the quality of X-ray optical elements for coherent applications. Although it is possible to select a transversely coherent portion with a pinhole, the beam size may be too small to characterize the optics. Propagation along a certain distance increases the transverse coherence length according to
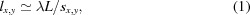
where *l* is the coherence length, λ is the wavelength, *L* is the distance from the source, and *s* is the source size. Based on this scheme, we built the 1 km beamline BL29XUL at SPring-8 and have used it extensively for coherence-related studies.

Typical transverse coherence lengths in the 1 km station at a wavelength λ = 1 Å are calculated to be *l*_*x*_ = 0.3 mm (FWHM) and *l*_*y*_ = 1.8 mm in the horizontal and vertical directions, respectively, with the current source parameters *s*_*x*_ = 316 µm and *s*_*y*_ = 4.9 µm in root mean square (r.m.s.) of SPring-8. The coherence lengths can be further increased by inserting an aperture in the optics hutch, which acts as a virtual source, at the expense of beam intensity. The beam size in the 1 km station is as large as 30 (H) mm × 10 (V) mm, which is suitable for the characterization and development of high-quality optical elements as shown below.

### 3.2. Be windows   

Many studies have noted the degradation of X-ray beam quality through beryllium windows. Roughness on the beryllium surface seemed to be the primary cause for degradation (Snigirev *et al.*, 1996 ▶). Although we initially used a polished beryllium window for the 1 km beamline, we could not suppress speckles. After systematic studies, we found that speckles with bright spots resulted from internal voids several to tens of micrometres in diameter for conventional beryllium foils fabricated by powder sintering (Electrofusion, PF-60) and high-purity ingot foil (Electrofusion, IF-1) (Goto *et al.*, 2004 ▶).

**Figure 3:**
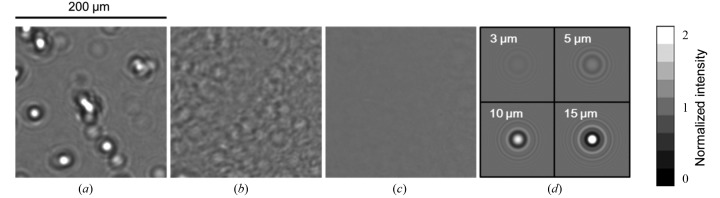
Transmission X-ray images through beryllium foils obtained at the 1 km beamline of SPring-8 at a wavelength of 0.1 nm: (*a*) powder foil (PF-60) of 250 µm thickness, (*b*) ingot foil (IF-1) of 250 µm thickness, and (*c*) PVD foil of 100 µm thickness. The distance between the beryllium foils and the detector is 1.4 m. (*d*) Calculated Fresnel diffraction patterns from spherical voids with diameters of 3, 5, 10 and 15 µm.

The third candidate tested was a beryllium foil fabricated using physical vapour deposition (PVD), which should have fewer or no internal voids (Goto *et al.*, 2007*b* ▶). Fig. 3 ▶ shows transmission X-ray images for PF-60, IF-1 and PVD beryllium foils measured at the 1 km beamline with a wavelength of 1 Å. They were supplied by Electrofusion and polished to 0.1 µm (r.m.s.) or less. A zooming tube (Hamamatsu) with a spatial resolution of 0.5 µm was used for observation. The sample–detector distance was set to 1.4 m. The Fresnel diffraction patterns calculated for spherical voids are shown in Fig. 3(*d*) ▶ with diameters ranging from 3 to 15 µm. The phase shift resulting from voids in the beryllium was −0.14 rad µm^−1^ for 0.1 nm X-rays, resulting in bright-spot diffraction. The experimental results show that PF-60 and IF-1 have voids of 10 µm and 5 µm diameter, respectively. The concentration of the voids, estimated to be 10^3^–10^4^ mm^−3^ for these materials (Goto *et al.*, 2004 ▶, 2007*b* ▶), is the primary cause for speckles when using conventional beryllium windows. PVD beryllium foils continue to be developed by manufacturers (Goto *et al.*, 2011 ▶), and they will be adopted as part of the speckle-free optics scheme for the DLSR.

A CVD diamond window is an alternative material for speckle-free windows. Advantages of the diamond window are larger tolerance for high heat load and reduced small-angle scattering (Jaski & Cookson, 2007 ▶). Although the Bragg diffractions from grains that accidentally satisfy the Bragg condition leave dark spots in the transmitted beam (Goto *et al.*, 2007*a* ▶), this problem could be mitigated by using, for example, nanocrystal diamond with a smaller grain size.

### 3.3. Reflective mirrors   

X-ray reflective mirrors are essential optical elements for designing various optical systems for X-ray focusing (Kirkpatrick & Baez, 1948 ▶), imaging (Matsuyama *et al.*, 2012*b* ▶) and interferometry. In particular, an ultra-precise figure shape is required for mirrors to control coherent wavefronts while suppressing unwanted speckles. The phase error ϕ of the wavefront in the reflected X-ray beam is expressed by
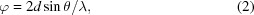
where *d* and θ are the height error and the incident angle, respectively. For the condition of θ = 5 mrad and λ = 0.8 Å, a height error of *d* = 8 nm generates a substantial phase error of ϕ = 1, which indicates that a high accuracy at the nanometre level is required to avoid deterioration of the wavefront. In addition, figure errors with a spatial period in the sub-millimetre region should be sufficiently suppressed in order to produce a uniform intensity profile (Mimura *et al.*, 2004 ▶; Yamauchi *et al.*, 2005 ▶). To achieve this level, several machining techniques have been developed, such as elastic emission machining (EEM) (Yamauchi *et al.*, 2002*a* ▶), ion beam figuring (Schindler *et al.*, 2001 ▶) and additional deposition (Ice *et al.*, 2000 ▶), as well as surface metrologies such as a long trace profiler (Takacs *et al.*, 1987 ▶) and stitching interferometers (Yamauchi *et al.*, 2003 ▶; Mimura *et al.*, 2005*a* ▶). Recently, one could achieve a high figure accuracy and small degree of roughness of 1 nm (peak to valley) and 0.2 nm (r.m.s.), respectively.

**Figure 4:**
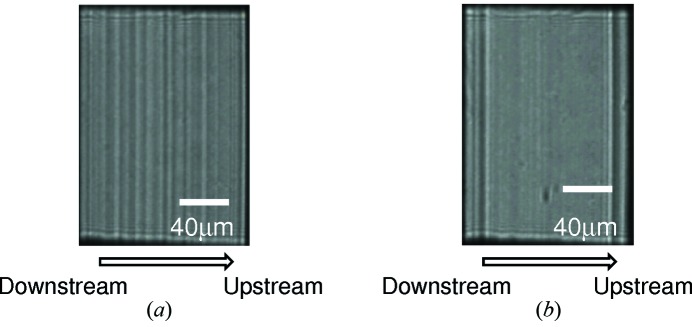
Reflected beam images from flat mirrors measured at the 1 km beamline of SPring-8 for (*a*) a non-EEM-processed area, and (*b*) an EEM-processed area. Photon energy is 15 keV, mirror length 70 mm, glancing incidence angle 1.2 mrad, incident slit width 100 µm, and distance between mirror centre and camera 166 mm.

In this subsection, we review high-quality mirrors developed at SPring-8. In 2001, we started to fabricate flat mirrors by combining EEM with a micro-stitching interferometer (MSI) (Yamauchi *et al.*, 2003 ▶). We achieved a flat intensity profile without speckles as shown in Fig. 4 ▶ (Mori *et al.*, 2001 ▶; Yamauchi *et al.*, 2005 ▶), measured at the 1 km beamline with a photon energy of 15 keV. The profiles agreed well with those expected from the measured surface figures and wave-optical simulations.

These techniques were further applied to the development of focusing mirrors with aspherical surfaces. We successfully fabricated an elliptical focusing mirror to generate nearly diffraction-limited focusing with a size of 200 nm. We also observed both constructive and destructive interference fringe patterns in the vicinity of the beam waist, which agreed with those produced by a wave-optical simulation (Yamauchi *et al.*, 2002*b* ▶). To produce tighter focusing with a large numerical aperture (NA), however, we needed to design a steeper curvature with a large incident angle. This condition posed a more severe requirement to surface metrology, as indicated in equation (2). For this purpose, we developed a relative angle determinable stitching interferometer (RADSI) (Mimura *et al.*, 2005*a* ▶), which enabled us to construct a two-dimensional focusing system with a beam size of 36 nm (vertical) × 48 nm (horizontal) in the KB geometry at a photon energy of 15 keV (Mimura *et al.*, 2005*b* ▶).

**Figure 5:**
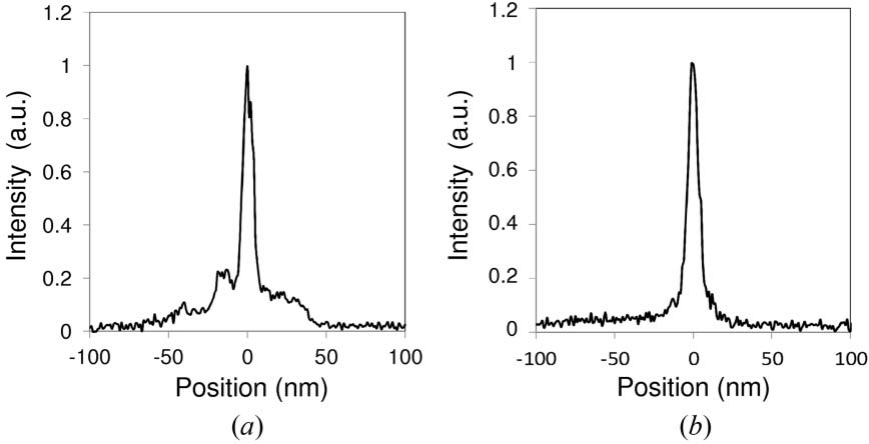
Sub-10 nm focused beam profiles along the (*a*) horizontal and (*b*) vertical direction, measured at the 1 km beamline of SPring-8 at a photon energy of 20 keV. Mirror lengths are 80 mm (horizontal) and 20 mm (vertical). The surfaces of the mirrors are coated with Pt/C multilayer.

A further increase of NA by using a graded multilayer coating allows for much smaller focus spots down to the sub-10 nm level. To achieve the higher accuracy required for this condition, we introduced adaptive optics, *i.e.* a deformable mirror that compensates for the excess error of the wavefront by applying a phase-retrieval method (Mimura *et al.*, 2010 ▶). We successfully generated a two-dimensional focused spot with a size of less than 10 nm at 20 keV, as shown in Fig. 5 ▶ (Yamauchi *et al.*, 2011 ▶).

Finally, we describe a two-stage focusing system, which consists of two KB focusing systems, originally developed for tight focusing of XFEL pulses. The first KB system serves to expand the beam size, while the second one generates a small focus with a large NA. We successfully generated ultra-intense X-ray pulses of 10^20^ W cm^−2^ with a size of 30 nm × 55 nm for 9.9 keV XFEL pulses from SACLA (Mimura *et al.*, 2014 ▶), which were applied to observe two-photon absorption for the germanium *K*-absorption edge of 11.1 keV (Tamasaku *et al.*, 2014 ▶). We note that this system is widely applicable for generating small spots for beamlines with a limited length. An extension of the system to variable focusing size is discussed in §5.2.

### 3.4. Diamond crystals   

High-pressure high-temperature (HPHT) synthetic type IIa diamond crystals (Burns *et al.*, 2009 ▶; Polyakov *et al.*, 2011 ▶; Sumiya & Tamasaku, 2012 ▶) are used for various synchrotron radiation applications due to their excellent thermal properties: low thermal expansion and high heat conductivity (300 K), high X-ray transmittance and radiation durability. Consequently, the diamond crystals will be used for the high-heat-load monochromator, beam splitter and phase plates at the DLSR.

**Figure 6:**
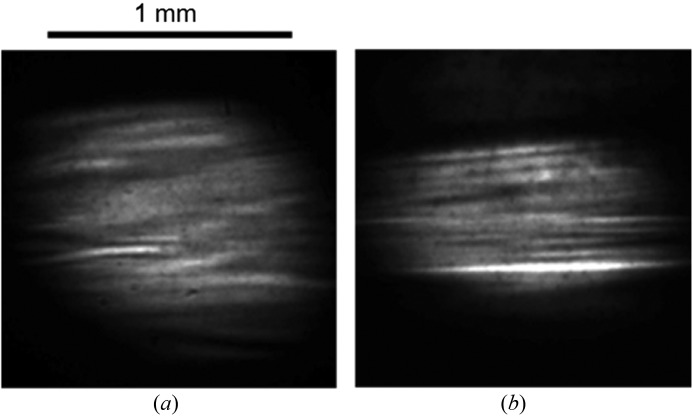
X-ray images of (111) reflections from the diamond double-crystal monochromator obtained at a distance of 11 m at a photon energy of (*a*) 7.74 keV and (*b*) 18.3 keV.

We characterized 〈001〉-growth IIa diamond crystals supplied by Sumitomo Electric Industries Ltd at the 1 km beamline (Tamasaku *et al.*, 2005 ▶) and they appeared to be almost perfect. The main limitation with the diamond crystals was the non-uniformity of the intensity profile of the reflected beam that was enhanced at the experimental station located at a distance over 10 m from the monochromator when they were used for the double-crystal monochromator (Yabashi *et al.*, 2007 ▶). A beam profile with speckles presents a serious problem for coherent X-ray applications. Fig. 6 ▶ shows X-ray beam images from the double-crystal monochromator at BL39XU of SPring-8. A non-uniform profile with fringes up to 50% (r.m.s.) was observed. We performed a simple simulation based on Fresnel diffraction from crystal segments, divided by the growth sector boundaries. When we assumed phase shifts due to a lattice inclination of 0.5 µrad or more, or a lattice step due to stacking faults, we could reproduce similar non-uniformity in the experimental results (Goto *et al.*, 2012 ▶). The simple model suggests that a smaller phase shift in the whole region in the crystal is essential for wavefront preservation. Diamond crystals with even better quality and a larger domain are required for practical use at DLSR facilities.

## 4. Diagnostics and stabilization   

As discussed in §2, DLSRs are capable of generating a small intense spot with a diameter of ∼100 nm and a photon flux of 10^14^ photons s^−1^ by direct demagnification of the source. Achieving this scheme requires high stability for the source and for every optical element especially in the reflection geometry, because one has to retract the aperture as the secondary source. The target level for angular stability is ∼0.1 µrad (equivalent to 10 µm at 100 m distance) in both the vertical and horizontal directions. This is a small fraction of the angular source size. The most demanding requirement is to stabilize the high-heat-load DCM. In §4.1 we describe research to improve the stability of DCMs. Another important step is to perform precise diagnostics of the beam properties. In §4.2, we present useful online diagnostics tools. In §4.3, we show diagnostics of higher-order coherence with Hanbury Brown and Twiss experiments in the X-ray region, which could provide access to ideal transverse coherence profiles and source sizes even under conditions of optical instability.

### 4.1. Stability improvement of DCM   

**Figure 7:**
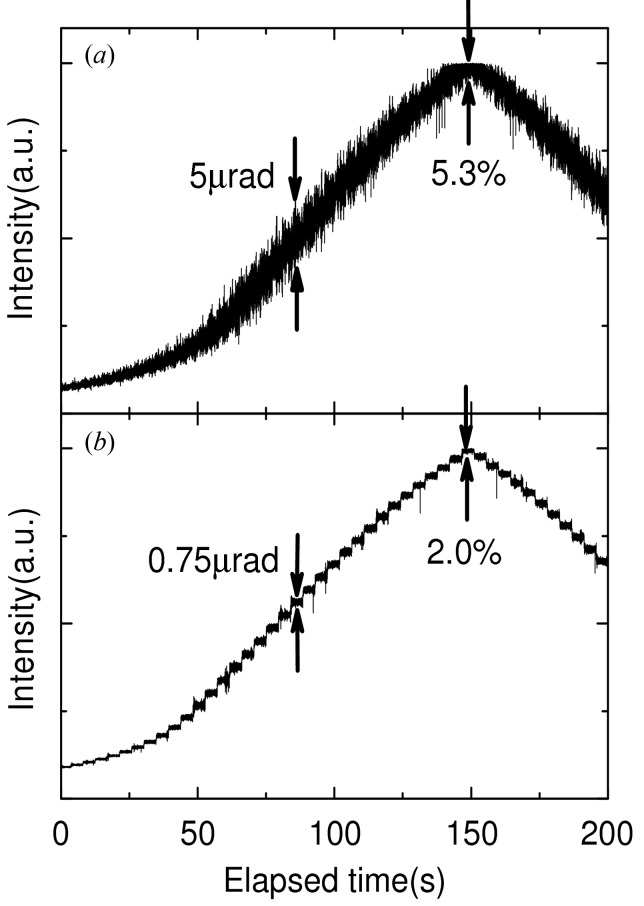
Time trace of the X-ray intensity transmitted by the LN_2_-cooled DCM. The first crystal was rotated with steps of 1 µrad at a time interval of 5 s. The LN_2_ flow channels for the two crystals inside the DCM are connected with (*a*) a standard flexible tube and (*b*) a low-vibration flexible tube.

Better stabilization of high-heat-load DCMs is one of the most critical challenges for effective utilization of DLSRs. In order to suppress the thermal deformation of silicon crystals, liquid-nitrogen (LN_2_) cooling has been widely applied to DCMs (Bilderback *et al.*, 2000 ▶; Mochizuki *et al.*, 2001 ▶). In this case, the bumping of vaporized nitrogen could be a cause of vibration, in addition to usual sources such as the pulsations of coolant pumps and the turbulence within flow channels. To suppress short-term fluctuations (*i.e.* vibrations), the characteristics of the flexible tubes used for the flow channel are very important (Yamazaki *et al.*, 2013 ▶), because the corrugation of the flexible tube can easily produce an unwanted turbulent flow of LN_2_. We recently developed a low-vibration flexible tube with low turbulence (patent pending jointly by RIKEN, JASRI and Osaka Rasenkan Kogyo Co. Ltd). Soft sleeves of alumina fibre are inserted into a standard flexible tube made of stainless steel to serve as a smooth lining. The inner tube remains pliable at LN_2_ temperatures under radiation. Fig. 7 ▶ shows time traces of the X-ray intensity downstream from the LN_2_-cooled DCM at a photon energy of 12 keV while scanning the deviation angle of the first crystal at a sampling rate of 1 kHz. Replacement of the standard tubes by flexible tubes resulted in a reduction of the angular fluctuation from 5 µrad to 0.75 µrad, corresponding to a decrease of the intensity fluctuation from 5.3% to 2.0%.

### 4.2. Online diagnostics   

Online photon diagnostics are a prerequisite for new light sources, not only for experiments but also for the stable operation of accelerators and beamlines. In many cases, only a small fraction of X-rays are sampled for diagnostics through the interactions between a transparent material and the X-rays. Such materials must possess the ‘speckle-free’ quality for wavefront preservation, as science applications highlighted by DLSRs rely on a high coherent flux, *e.g.* coherent X-ray imaging and X-ray photon correlation spectroscopy. For the in-line photon diagnostic systems of DLSRs, good candidates are found in XFEL beamlines, which also require the preservation of coherent wavefronts. This subsection introduces in-line intensity and profile diagnostics that have been in operation at SACLA (Ishikawa *et al.*, 2012 ▶; Tono *et al.*, 2013 ▶).

**Figure 8:**
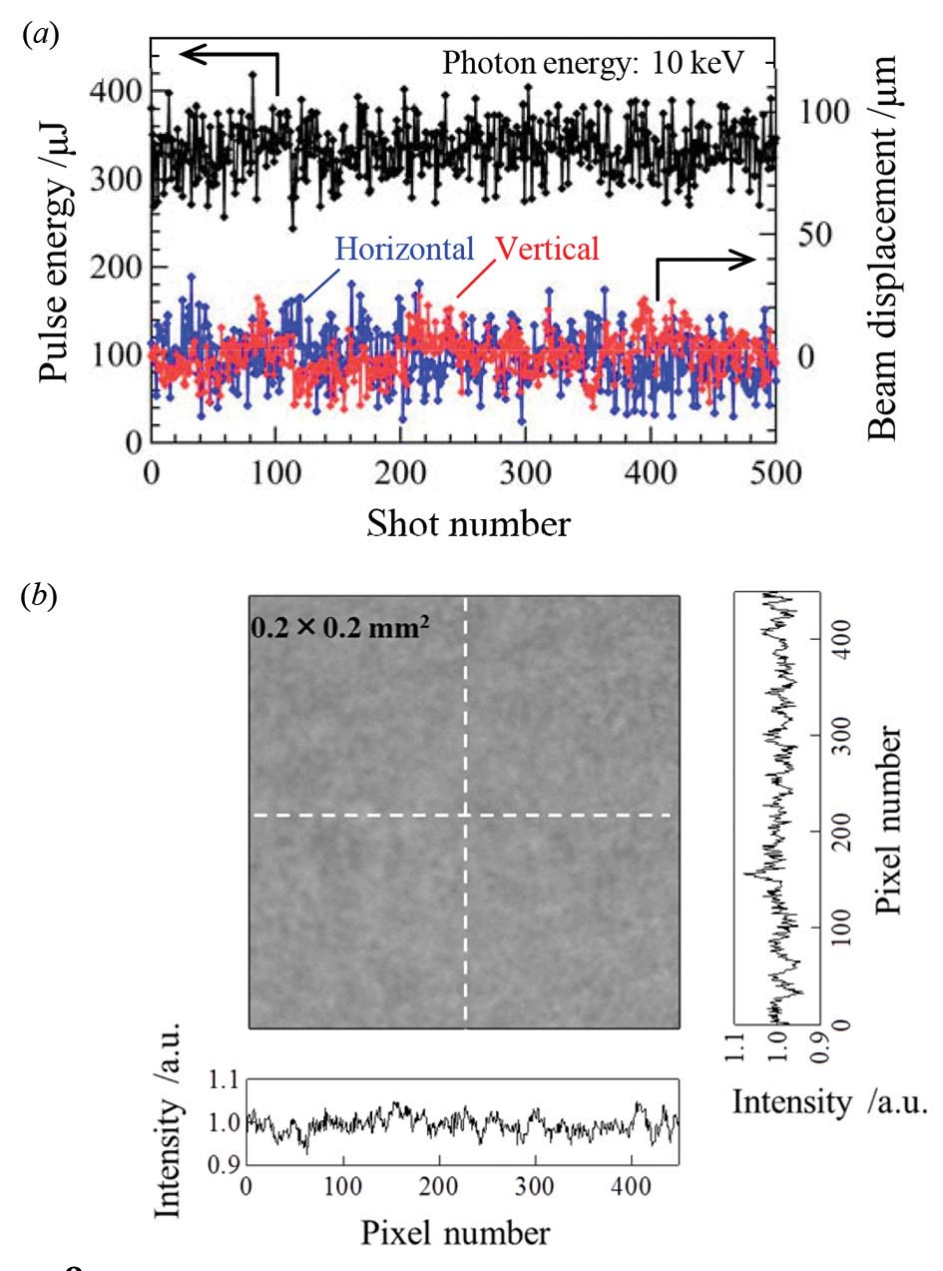
(*a*) Trend plot of XFEL pulse energies (left axis) and positions (right axis) measured with the monitor in a pulse-by-pulse manner. A pulse energy of 100 µJ corresponds to 6 × 10^10^ photons per pulse. (*b*) Transmitted coherent X-ray image of the nanocrystal diamond foil in the monitor. Signal intensities over the 450 × 450 pixels have a standard deviation of only 2% of the average. Cross-sectional profiles along the dotted lines are shown.

The first example is an in-line intensity/position monitor, in which scattered X-rays from a thin foil are detected with quadrant photodiodes (Alkire *et al.*, 2000 ▶; Tono *et al.*, 2011 ▶). The foil, only 15 µm thick, is composed of small diamond crystals with an average grain size of a few tens of nanometres. The total signal intensity of the four photodiodes is proportional to the pulse energy of XFEL light. This measurement principle necessitates calibration to provide the absolute pulse energy. For this purpose, an absolute intensity monitor for XFELs is currently available [*i.e.* a radiometer and a gas monitor detector (Kato *et al.*, 2012 ▶)]. The beam position is obtained from the ratio of the difference to the sum of the intensities measured by the horizontal and vertical pairs of photodiodes. For example, a beam displacement by Δ*x* in the horizontal direction is found from
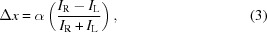
where *I*_R_ and *I*_L_ are the signal intensities of the right and left photodiodes, respectively, and α is a proportionality factor. Fig. 8(*a*) ▶ shows a trend plot of pulse energies and beam positions measured in a shot-by-shot manner at photon energy of 10 keV. Small errors of the position measurement, being sufficiently less than one-tenth of the beam size of ∼300 µm, would be promising for accurate monitoring of X-ray beams from DLSRs.

A thin diamond foil is also applied to the diagnostics of the spatial profile. It is well known that diamonds with impurities and/or defects emit fluorescence in the visible-light wavelength range. Chemical vapour deposition (CVD) provides impurity-doped diamond foils with well controlled impurity concentrations. The diagnostic system of SACLA includes 30 µm-thick foils of boron-doped CVD diamonds which emit fluorescence in the visible spectrum. The surface of each foil was polished to have an average roughness less than 50 nm. This level of surface quality is sufficient for avoiding deterioration of the coherent wavefront of XFEL light.

The above monitors owe their ability for online measurement to the high X-ray transmittance and speckle-free quality of the diamond foils, as evaluated at the 1 km beamline (see §3.1 and §3.2) (Goto *et al.*, 2007*a* ▶). Fig. 8(*b*) ▶ shows a transmission coherent-X-ray image of the nanocrystal diamond foil for the intensity/position monitor. This test indicated the absence of significant voids, impurities and surface roughness in the foil. The speckle-free quality has been successfully demonstrated in operations at SACLA.

### 4.3. Diagnostics of second-order coherence and applications   

For chaotic sources such as synchrotron light sources, transverse coherence length is reciprocally proportional to the source size, as seen in equation (1). The coherence length can be deteriorated by possible instability of the source and/or optics, which enlarges the effective source size seen from the sample position. However, the second-order coherence measurement, first proposed by Hanbury Brown and Twiss (HBT), is free from such fluctuation and instability, because the characteristic time to measure the coherence is determined by the time window of the coincidence circuit for CW sources (Hanbury Brown & Twiss, 1956*a* ▶,*b* ▶) or by the pulse duration for pulsed sources including synchrotrons (Ikonen, 1992 ▶). Comparison with usual first-order coherence measurements such as a Young’s double slit provides a good indication of the stability level and its influence on coherence.

**Figure 9:**
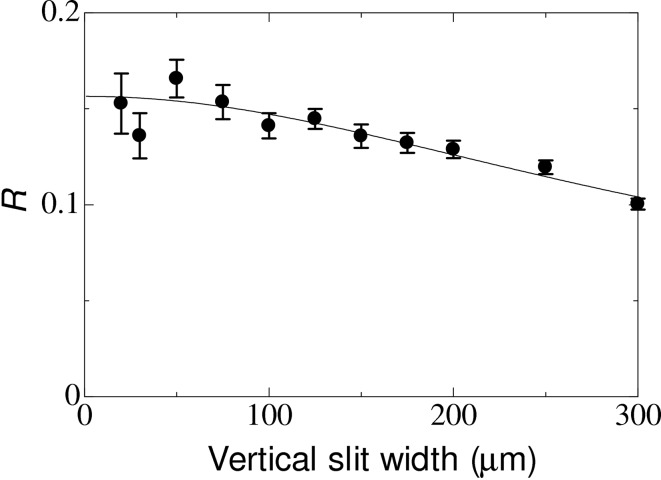
Excess coincidence ratio *R* as a function of vertical slit width. The solid circles are experimental data, while the solid curve is the best fit for a Gaussian coherence profile with a coherence length of 161.3 µm.

Fig. 9 ▶ shows the result of an X-ray HBT experiment to determine the transverse coherence length and the source size (Yabashi *et al.*, 2001*a* ▶, 2004 ▶), performed at BL29XUL of SPring-8. Here the excess ratio *R* of the coincidence is plotted as a function of the vertical slit width. Since *R* is given by the ratio of the temporal coherence time to the pulse duration, a high-resolution X-ray monochromator, which features four highly asymmetric reflections and has a bandwidth of 120 µeV at a photon energy of 14.4 keV, was used to enhance *R* (Yabashi *et al.*, 2001*b* ▶). From the measured dependency of *R* on the vertical slit width, the coherence length at a distance of 53.3 m from the source was determined to be 161.3 ± 5.0 µm, which corresponds to a vertical source size and a vertical emittance of 4.5 ± 0.1 µm and 3.6 ± 0.2 pm rad, respectively. These values agreed well with predictions. The HBT experiments will provide accurate and useful information on coherence and source properties for DLSRs.

## 5. New experimental schemes with innovative optics   

In this section we discuss new experimental opportunities using innovative X-ray optics. First we introduce two-dimensional focusing with a single mirror element. Second is an application of adaptive optics to enable variable focusing size while maintaining the focus position, which facilitates combinative analyses in an end-station. Last is a new optical scheme for emission spectroscopy, which combines a parabolic mirror and crystal optics.

### 5.1. Two-dimensional focusing using a single mirror element   

Although focusing systems that use a single mirror, such as an ellipsoidal mirror or a Wolter mirror, promise high potential for nano-focusing, it has been difficult to fabricate them due to their complicated and extremely steep profiles. However, recent breakthroughs with precision machining technology have enabled their development (Motoyama *et al.*, 2014 ▶). As a prototype, we have fabricated an ellipsoidal focusing mirror that has a ring-shaped aperture for the water-window wavelength region (Takei *et al.*, 2013 ▶). The fabrication system consists of mandrel fabrication, surface replication, metrology and refiguring. An objective for the mirror is to focus soft X-ray FEL light to the sub-10 nm region without chromatic aberration (Motoyama *et al.*, 2013 ▶).

For hard X-ray ranges, a grazing-incidence ellipsoidal mirror, which has a very steep curve in the transverse direction to the incident X-rays, is also under development. An advanced stitching interferometer was developed for profiling the three-dimensional shape by combining MSI and RADSI (Yumoto *et al.*, 2010 ▶). An upgraded EEM (Takei & Mimura, 2014 ▶) system will also be used for fabrication.

### 5.2. Variable-sized focus using adaptive optics   

Recent developments in X-ray focusing devices have enabled great progress in nano-analysis using focused beams. Compared with electron microscopes using an electromagnetic lens, however, combinative analyses with flexible focusing size remain challenging, because it is more difficult to change the optical parameters in an X-ray focusing system. As a result, optical systems for high-resolution scanning X-ray microscopy with a small nano-beam are usually constructed independently from instruments for coherent diffraction microscopy with more modest focusing. Although tuning of the focus size is possible by changing the size of the aperture served as a virtual source, the loss of photon flux, especially for smaller foci, is significant.

An adaptive focusing device (Susini *et al.*, 1995 ▶) is a promising candidate for expanding flexibility. Recently, we achieved a ∼100 nm focus using piezoelectric deformable mirrors with bimorph structures at SPring-8 (Nakamori *et al.*, 2013 ▶). We could precisely control the shape of a deformable mirror by using techniques for the at-wavelength wavefront measurement such as grating interferometry (Matsuyama *et al.*, 2012*c* ▶) and the pencil beam method (Hignette *et al.*, 1997 ▶).

**Figure 10:**
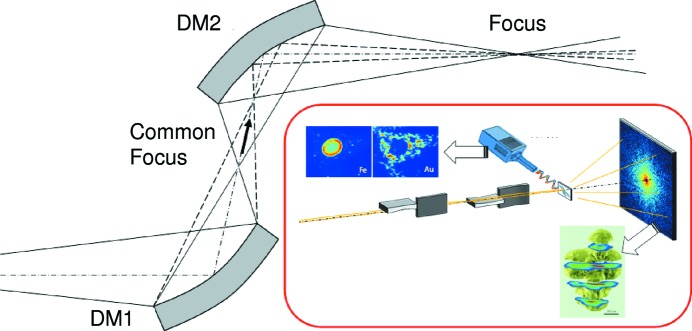
Schematic of a one-dimensional adaptive focusing system. NA and final focus size are controlled by changing the figures of the two deformable mirrors (DMs). The inset shows an example of coherent diffraction imaging (CDI) and scanning microscopy.

A drawback of the single adaptive element applied for the diffraction-limited focusing is the change of the focus position along the optical axis. To overcome this problem, we propose an innovative adaptive focusing optical system comprising four deformable mirrors arranged in a two-stage KB configuration, as shown in Fig. 10 ▶ (Matsuyama *et al.*, 2012*a* ▶; Kimura *et al.*, 2013 ▶). This optical system can control the spot size at a fixed sample position while maintaining the diffraction-limited focusing, by controlling the numerical aperture. We plan to use this system to deliver coherent X-rays with a controllable beam size ranging from 1 µm to a few tens of nanometres for various types of microscopy.

### 5.3. Optics for emission spectroscopy with parallel beam geometry   

Advances in fabricating aspherical mirror surfaces with unprecedented precision open up new optical configurations, *e.g.* a mirror-based spectrometer for various types of emission spectroscopy measurements including inelastic X-ray scattering (Schülke, 2007 ▶) and X-ray nonlinear optics.

**Figure 11:**
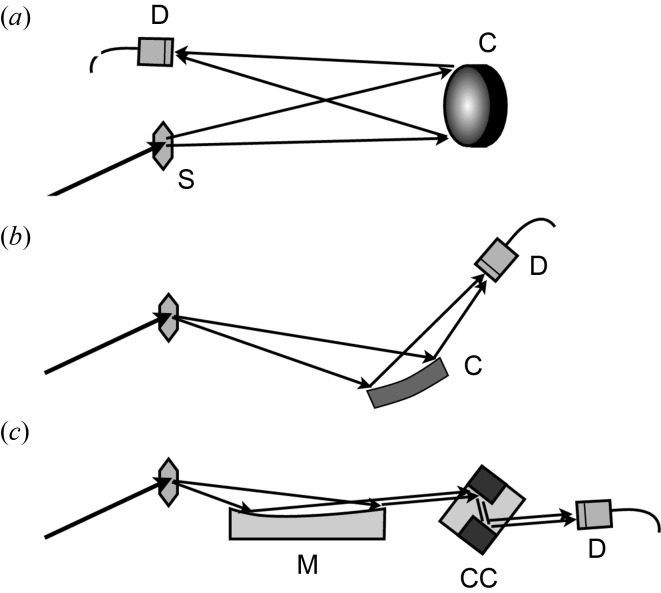
Spectrometer design for divergent X-rays. The conventional set-up uses (*a*) spherically and (*b*) cylindrically bent crystals. (*c*) A parabolic mirror collimates the beam prior to entering a channel-cut monochromator. S: sample, C: crystal, CC: channel-cut crystal, M: mirror, D: detector.

Conventionally, cylindrically or spherically bent crystals have been widely used in the Rowland or von Hamos geometries to disperse divergent X-rays from samples [Figs. 11(*a*) and 11(*b*) ▶]. However, the curvature radius tends to become longer in order to avoid distortion, which enlarges the size of the spectrometer to, for example, ∼10 m length. Furthermore, the optical layout is quite limited. For example, multicrystal optics, which is widely used in X-ray monochromators to control the bandwidth, cannot be applied. Now it becomes possible to collimate the divergent beam with aspherical mirrors. Once the beam is made parallel, the spectrometer can be designed more flexibly. A multicrystal spectrometer may be used to achieve sub-meV resolution. Mirror-based spectrometers may become even more compact.

Here, we present a prototypical mirror-based spectrometer, which consists of a parabolic mirror and a channel-cut analyser crystal (Fig. 11*c* ▶), designed for future X-ray parametric down-conversion experiments (Tamasaku *et al.*, 2011 ▶). In this nonlinear optical phenomenon, a down-converted X-ray photon is emitted into a small solid angle of, for example, 1 × 10^−5^ sr, and has a typical bandwidth of about 1 eV at ∼10 keV. The parabolic mirror is 400 mm long with an effective width of 4 mm, and is manufactured on a fused silica block. The surface is coated with a Pt film with a thickness of 60 nm. The roughness and the figure error are measured to be 0.12 nm (r.m.s.) and 0.7 nm (r.m.s.), respectively. The distance between the sample and the mirror centre is designed to be 400 mm, and the glancing angle at the centre is 4 mrad. Thus, the acceptance angle of the mirror is 1.6 mrad within the reflecting plane and 10 mrad along the surface. A channel-cut Ge(220) monochromator is set just downstream from the mirror.

**Figure 12:**
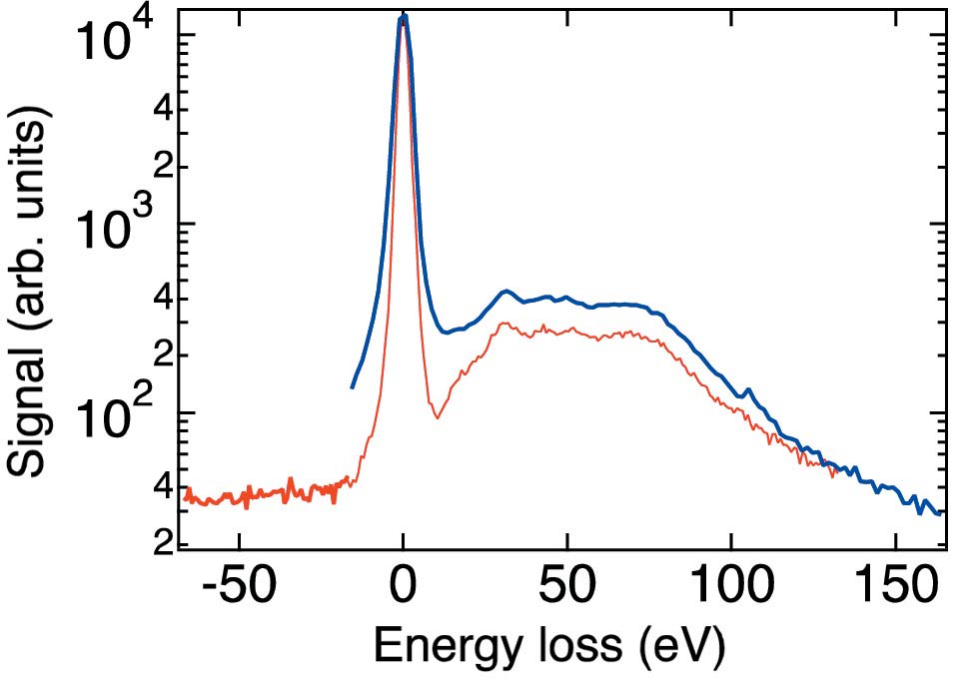
Energy spectra of X-rays scattered by a diamond sample. The pump photon energy is 8 keV. The red and blue lines are measured by the mirror-based and the bent-crystal spectrometers, respectively.

Fig. 12 ▶ shows the spectrum of the scattered X-rays from a diamond sample measured at BL19LXU in SPring-8. The pump photon energy is 8 keV. The peak at the origin is the Rayleigh line and the broad peak around 50 eV is due to Compton scattering. We also plot a spectrum measured with a conventional spectrometer, which consists of a cylindrically bent Ge(220) monochromator set 1.5 m away from the sample (Fig. 11*b* ▶). Compared with a conventional one, the mirror-based spectrometer shows better performance, revealing sharper structures, especially near the strong elastic peak. The results provide clear evidence for the advantages of the use of an aspheric mirror.

The prototypical mirror-based spectrometer can be easily upgraded using the KB set-up to collimate the beam two-dimensionally for higher energy resolution. To cover a larger solid angle, a multilayer coating can be used, although the photon-energy range must be fixed. Such innovative spectrometers are fully compatible with the beam characteristics of DLSRs, which allows a smaller focus size on the sample and a higher photon flux with a narrower bandwidth.

## Figures and Tables

**Table 1:** Comparison of electron beam parameters of SPring-8 II (tentative) and SPring-8

	SPring-8 II	SPring-8
Energy (GeV)	6	8
Unit-cell structure	5 bending magnets	2 bending magnets
Ring structure	2 injection cells + 42 unit cells + 4 straight cells	44 unit cells + 4 straight cells
Length of insertion-device straight (m)	4.684	6.65
Natural emittance (nm rad)	0.15 (achromat)	2.4 (non-achromat)
	∼0.10 (achromat)†	
Coupling ratio (%)	10	0.2
Beam sizes (σ_*x*_, σ_*y*_) at insertion device (µm)	24.0, 5.6	316, 4.9

† Including radiation damping effect with closing undulator gaps.
